# Sperm Cryopreservation Activity and Quality Assurance of Cryogenic Storage at a University Hospital in Oujda, Morocco

**DOI:** 10.7759/cureus.113745

**Published:** 2026-07-31

**Authors:** Boutayna Elrhaffouli, Dounia El Moujtahide, Hicham Ezzaidi, Elhoucine Sebbar, Mohammed Choukri

**Affiliations:** 1 Biochemistry, Mohammed VI University Hospital, Oujda, MAR

**Keywords:** cryogenic storage, fertility preservation, liquid nitrogen, male infertility, quality assurance, sperm cryopreservation

## Abstract

Background

Sperm cryopreservation is the standard method for preserving male fertility in patients at risk of impaired reproductive function. As the demand for fertility preservation increases, maintaining optimal cryogenic storage conditions through robust quality assurance programs has become essential to ensure the long-term safety of cryopreserved specimens.

Objective

The primary objective was to evaluate the quality assurance program for cryogenic storage at the Assisted Reproductive Technology and Gamete Cryopreservation Unit of Mohammed VI University Hospital in Oujda, Morocco. As a complementary objective, we described the unit's sperm cryopreservation activity and clinical indications to contextualize the biological specimens whose long-term safety depends on this storage system.

Methods

A retrospective descriptive and analytical study was conducted between March 2022 and November 2025. Fifty-nine sperm cryopreservation procedures were analyzed. Semen parameters were analyzed according to the World Health Organization (WHO) recommendations. Clinical indications were classified as oncological or non-oncological. Comparisons were performed using the Mann-Whitney U and Kruskal-Wallis tests, while correlations were assessed using Spearman's rank correlation coefficient. Additionally, 381 liquid nitrogen monitoring records were analyzed to assess storage stability, evaporation rates, refill frequency, seasonal variations, and temporal trends in cryogenic storage performance.

Results

The mean patient age was 31.1±9.2 years. Oncological indications predominated, with testicular tumors representing the most frequent indication, followed by hematological malignancies. Patients with oncological indications were significantly younger than those with non-oncological conditions (p<0.001) and differed significantly in sperm concentration (p=0.028) and the number of cryopreserved straws (p=0.015). A positive correlation was observed between sperm concentration and motility (ρ=0.373; p=0.004). Monitoring of cryogenic storage demonstrated excellent storage stability, with no breach of the cryogenic storage chain or loss of biological specimens during the study period. Seasonal analysis revealed significantly higher liquid nitrogen evaporation rates during the warm season for one storage tank (p=0.040), while annual evaporation rates varied significantly over the four-year period (p<0.001).

Conclusion

At our institution, sperm cryopreservation was used for both oncological and non-oncological indications. Over the study period, structured monitoring and preventive refilling maintained safe storage conditions without a recorded breach of the cryogenic chain or specimen loss, despite seasonal and temporal variation in liquid nitrogen consumption. These institution-specific findings support continued rigorous monitoring but require confirmation in larger, multicenter studies.

## Introduction

Sperm cryopreservation is currently considered the standard method for male fertility preservation. Storage of spermatozoa in liquid nitrogen at -196°C allows long-term preservation of reproductive potential while maintaining gamete fertilizing capacity. Indications include both oncological and non-oncological conditions: chemotherapy, radiotherapy, and some surgical procedures may impair spermatogenesis, while severe oligozoospermia, semen collection difficulties, testicular disorders, and situations associated with a risk of secondary azoospermia may also justify preventive preservation [1-7].

The growing use of sperm cryopreservation requires rigorous management of cryogenic storage systems. Sample safety depends on maintaining adequate liquid nitrogen levels, ensuring refill traceability, anticipating supply needs, and routinely monitoring storage tanks. These elements are key indicators of quality assurance in sperm banks [2,8].

Clinical cryopreservation activity and cryogenic storage quality assurance are operationally linked: the clinical indications and volume of activity define the specimens and workload that the storage system must safely accommodate. Nevertheless, few studies have evaluated these two components together, particularly in low- and middle-income countries.

Accordingly, the primary objective of this study was to evaluate the quality assurance procedures for cryogenic storage at Mohammed VI University Hospital in Oujda, Morocco, by assessing liquid nitrogen levels, evaporation, refill frequency, and storage tank monitoring over nearly four years. The secondary objective was to describe sperm cryopreservation activity and clinical indications during the same institutional period, thereby placing the storage quality findings in their clinical context.

## Materials and methods

Study design, setting, and population

This retrospective descriptive and analytical study was conducted at the Assisted Reproductive Technology and Gamete Cryopreservation Unit of Mohammed VI University Hospital in Oujda, Morocco, after obtaining approval from the Biomedical Research Ethics Committee of the Faculty of Medicine and Pharmacy of Oujda, Mohammed First University (approval number: 10/24). The analysis included all men who underwent sperm cryopreservation between March 2022 and November 2025. All patients referred for male fertility preservation during the study period were eligible for inclusion, and a total of 59 sperm cryopreservation procedures were analyzed.

Indications for sperm cryopreservation were classified as oncological or non-oncological. Oncological indications included patients scheduled to receive potentially gonadotoxic treatments such as chemotherapy, radiotherapy, or surgery; these included testicular cancer, lymphoma, acute lymphoblastic leukemia, Ewing sarcoma, germ cell tumors, nasopharyngeal carcinoma, and other malignancies. Non-oncological indications included severe oligozoospermia, progressive deterioration of semen quality, varicocele, testicular ectopia, testicular torsion, retrograde ejaculation, inguinal fistula, inflammatory bowel disease, and other clinical conditions associated with an increased risk of impaired fertility.

Semen collection, analysis, and cryopreservation procedure

Semen samples were obtained by masturbation after a recommended period of sexual abstinence ranging from two to seven days. Samples were collected in sterile containers and allowed to liquefy for approximately 30 minutes at room temperature before analysis. Semen analysis was performed according to the recommendations of the World Health Organization Laboratory Manual for the Examination and Processing of Human Semen, Sixth Edition. The recorded variables comprised patient age, ejaculate volume, sperm concentration, total sperm motility, number of cryopreserved straws, and clinical indication for cryopreservation. Sperm concentration was determined after appropriate dilution using a counting chamber under light microscopy, while sperm motility was assessed by microscopic examination and expressed as the percentage of motile spermatozoa.

Following semen analysis, samples were gradually mixed with one of the commercial cryoprotective media available through routine laboratory procurement during the study period (SpermaFreeze® (FertiPro NV, Beernem, Belgium), ArcticSperm® (Nidacon International AB, Gothenburg, Sweden), or SperCryo All Round® (Cryo BioSystem, L'Aigle, France)). Product selection was therefore based on availability rather than on patient or semen characteristics. Although the commercial product varied, a standardized laboratory workflow was maintained throughout the study period: each medium was prepared and mixed with the semen sample using the ratio, equilibration conditions, and handling steps specified by its manufacturer; the mixture was then aliquoted into individually labelled cryogenic straws and subjected to the same controlled-rate cooling and long-term storage process in liquid nitrogen at -196°C. The number of cryopreserved straws prepared for each patient depended on semen volume and sperm quality.

Cryogenic storage quality assurance

The quality assurance component evaluated the cryogenic storage system used for gamete preservation within the same unit. Liquid nitrogen monitoring records collected between February 2022 and November 2025 were reviewed. Cryopreserved specimens were stored in liquid nitrogen tanks maintained at -196°C. The laboratory operated four storage compartments: Tanks 21/A and 21/B, which served as primary long-term storage tanks, and Tanks 26/A and 26/B, which served as working tanks. The primary tanks were maintained close to maximum capacity to ensure sample safety, whereas the working tanks were opened and refilled more frequently, explaining their greater variability.

**Figure 1:**
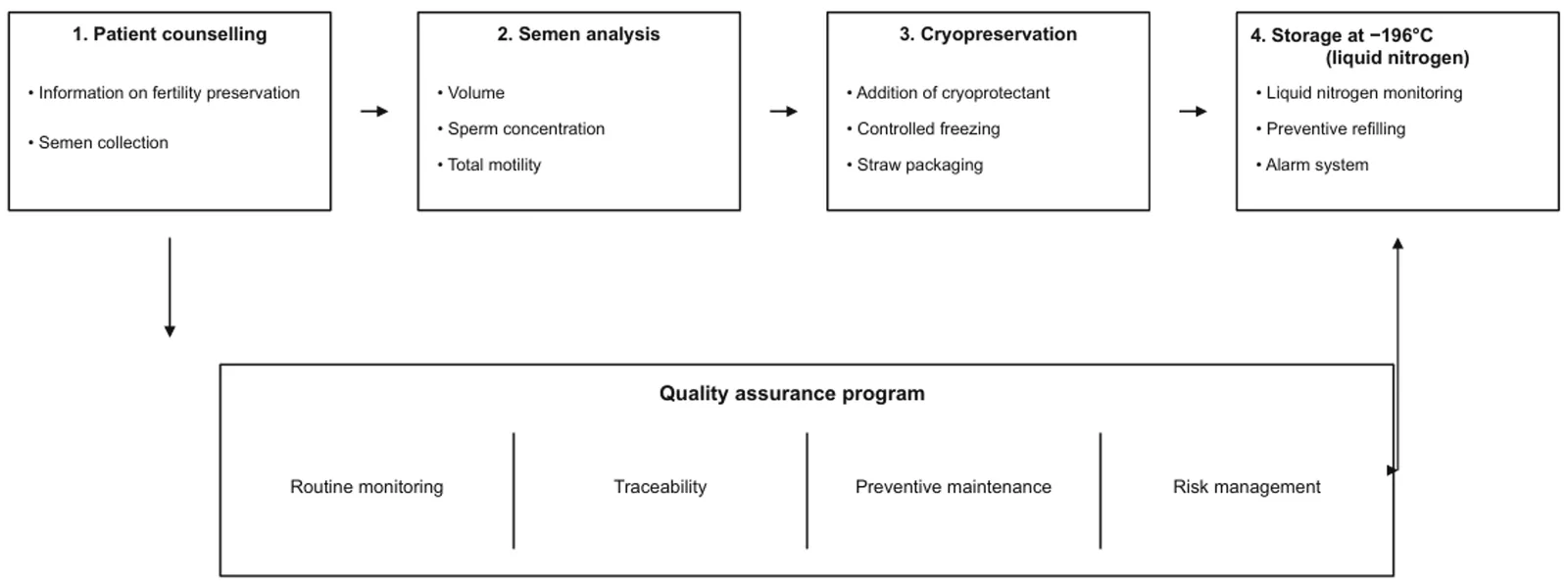
Workflow of sperm cryopreservation and quality assurance of cryogenic storage The figure illustrates the successive steps of the sperm cryopreservation process, including patient counselling, semen analysis, cryopreservation, and long-term storage in liquid nitrogen. The quality assurance program encompasses regular monitoring of liquid nitrogen levels, traceability, preventive maintenance, and continuous quality control to ensure the safety and integrity of cryopreserved specimens.

For each monitoring event, the date of inspection, liquid nitrogen level in each storage tank, refill operation, and liquid nitrogen delivery were recorded in dedicated monitoring logs. Routine manual level checks were scheduled two to three times per week and were repeated after refilling to verify restoration of the operating level. Refill decisions were guided by the predefined operating range of each tank: primary storage tanks were maintained close to maximum capacity, whereas working tanks were refilled when their level approached the lower operating threshold; any value below five units was classified as critical and required prompt corrective refilling. Each intervention was documented to preserve traceability. The evaluated indicators included the total number of monitoring controls, mean liquid nitrogen level, minimum and maximum levels, standard deviation, annual evolution of nitrogen levels, refill frequency, storage tank stability, and occurrence of critical levels. Figure 1 summarizes the sperm cryopreservation workflow and the main quality assurance steps for cryogenic storage.

Statistical analysis

Statistical analyses were performed using IBM SPSS Statistics for Windows, Version 25.0 (IBM Corp., Armonk, New York, United States) and Microsoft Excel (Microsoft Corporation, Redmond, Washington, United States). Continuous variables were expressed as mean±standard deviation, median, minimum, maximum, and interquartile range when appropriate, whereas categorical variables were presented as frequencies and percentages. Because several semen parameters were not normally distributed, non-parametric tests were used. Comparisons between oncological and non-oncological indications, as well as between patients with testicular cancer and those with other malignancies, were performed using the Mann-Whitney U test. Changes in semen characteristics according to year were assessed using the Kruskal-Wallis test, and associations between quantitative variables were evaluated using Spearman's rank correlation coefficient.

For cryogenic storage monitoring, temporal changes in liquid nitrogen levels were analyzed throughout the study period. Daily evaporation rates were calculated from consecutive monitoring records after the exclusion of measurements obtained immediately after refilling. Seasonal differences were evaluated by comparing the warm season, defined as May to September, with the cold season, defined as October to April, using the Mann-Whitney U test. A two-sided p-value of <0.05 was considered statistically significant.

## Results

General characteristics of the study population

A total of 59 sperm cryopreservation procedures were included in the study. The mean age of the patients was 31.1±9.2 years, with a median age of 30 years and a range from 19 to 57 years. The mean ejaculate volume was 1.75±1.03 mL, with a median of 1.5 mL and a range from 0.3 to 5.5 mL. The mean sperm concentration was 21.0±30.1 million spermatozoa/mL, while the median concentration was 7.5 million/mL, with values ranging from 0.4 to 133 million/mL. The mean total sperm motility was 30.3±19.5%, with a median value of 30.6% and a range from 0% to 84%. The mean number of cryopreserved straws was 3.93±2.11, with a median of four straws and a range from one to 11 straws.

Indications for sperm cryopreservation

Oncological indications accounted for the majority of sperm cryopreservation procedures, representing 36 of the 59 cases (61%). Among malignant diseases, testicular tumors were the most frequent indication, followed by lymphoma, acute lymphoblastic leukemia, germ cell tumors, Ewing sarcoma, nasopharyngeal carcinoma, and rectal adenocarcinoma. Non-oncological indications accounted for 23 cases (39%), with severe oligozoospermia and assisted reproduction or semen collection difficulties being the most common reasons for preservation. The detailed distribution is presented in Table 1. Percentages were calculated using all 59 procedures as the denominator; category totals clarify the distribution of oncological and non-oncological indications.

**Table 1:** Distribution of sperm cryopreservation procedures according to clinical indications

Category	Clinical indication	n (%)
Oncological	Testicular tumors	15 (25.4)
	Lymphomas	6 (10.2)
	Acute lymphoblastic leukemia	4 (6.8)
	Nasopharyngeal carcinoma	3 (5.1)
	Ewing sarcoma	2 (3.4)
	Rectal adenocarcinoma	2 (3.4)
	Other malignancies	4 (6.8)
Non-oncological	Severe oligozoospermia	8 (13.6)
	Assisted reproduction/collection difficulties	6 (10.2)
	Inguinal fistula at risk of azoospermia	3 (5.1)
	Testicular torsion/testicular ectopia	2 (3.4)
	Surgery associated with infertility risk	2 (3.4)
	Retrograde ejaculation	1 (1.7)
	Inflammatory bowel disease	1 (1.7)
Total oncological indications		36 (61)
Total non-oncological indications		23 (39)
Overall		59 (100)

Comparison between clinical groups and correlation analysis

Patients referred for oncological indications were significantly younger than those with non-oncological indications (p<0.001). A statistically significant difference was also observed in sperm concentration (p=0.028), indicating distinct semen profiles between the two groups. Similarly, the number of cryopreserved straws differed significantly according to the indication for cryopreservation (p=0.015). No statistically significant differences were found regarding ejaculate volume (p=0.138) or sperm motility (p=0.108).

Among patients with malignant diseases, only age differed significantly between men with testicular tumors and those with other cancers (p=0.027). No significant differences were observed in ejaculate volume (p=0.648), sperm concentration (p=0.981), sperm motility (p=0.981), or the number of cryopreserved straws (p=0.553). Across the 2022-2025 study period, no statistically significant annual differences were observed for ejaculate volume (p=0.560), sperm motility (p=0.498), sperm concentration (p=0.061), or number of cryopreserved straws (p=0.792), although sperm concentration showed a trend toward statistical significance over time.

Spearman's correlation analysis demonstrated a weak but statistically significant positive correlation between patient age and sperm concentration (ρ=0.272; p=0.037). No significant correlation was found between age and sperm motility (ρ=0.054; p=0.682). Conversely, a moderate positive correlation was observed between sperm concentration and sperm motility (ρ=0.373; p=0.004), indicating that higher sperm concentrations were associated with better sperm motility.

Cryogenic storage monitoring and liquid nitrogen levels

**Figure 2:**
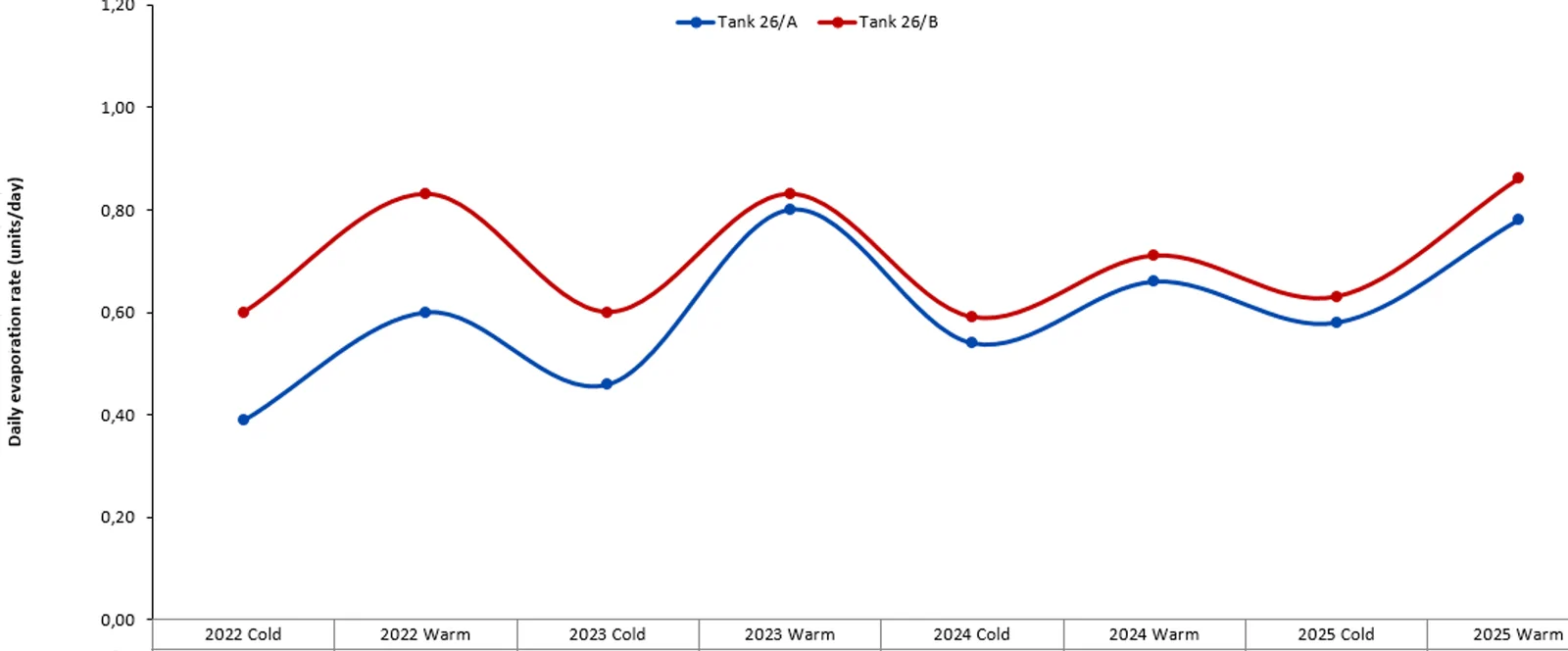
Temporal evolution of daily liquid nitrogen evaporation rates in storage Tanks 26/A and 26/B between 2022 and 2025 Daily evaporation rates are presented according to year and season. Blue shaded areas represent the cold season (October-April), whereas orange shaded areas indicate the warm season (May-September). A significantly higher evaporation rate was observed during the warm season for Tank 26/B (Mann-Whitney test; p=0.040), while no significant seasonal difference was found for Tank 26/A (p=0.608). Values are expressed as liquid nitrogen loss (units/day).

Between February 2022 and November 2025, 381 liquid nitrogen level measurements were performed to monitor the cryogenic storage system. Monitoring was regular throughout the study period, with 85 controls in 2022, 107 in 2023, 110 in 2024, and 79 in 2025. During the same period, 347 refill operations were performed, including 76 in 2022, 101 in 2023, 102 in 2024, and 68 in 2025. This corresponded to an average monitoring frequency of two to three inspections per week, supporting the continuous surveillance of storage conditions (Figure 2).

Storage Tanks 21/A and 21/B maintained highly stable nitrogen levels throughout the study, with mean values of 20.88±0.97 and 20.71±1.57 units, respectively. Greater variability was observed in Tanks 26/A and 26/B, with mean levels of 7.01±5.61 and 8.28±5.74 units, respectively. Maximum nitrogen levels reached 21 units in both primary storage tanks, reflecting regular refill procedures and effective maintenance of optimal storage conditions. The wider fluctuations observed in the working tanks corresponded to normal nitrogen consumption cycles and scheduled refill operations, as detailed in Table 2.

**Table 2:** Characteristics and stability of liquid nitrogen storage tanks during the study period

Storage tank	Mean±SD	Median	Range (min-max)	Interpretation
21/A	20.88±0.97	21	10.5-21	Primary long-term storage tank; stable level
21/B	20.71±1.57	21	10-21	Primary long-term storage tank; stable level
26/A	7.01±5.61	7	0-21	Working tank; variability related to consumption and refill cycles
26/B	8.28±5.74	9	0-20	Working tank; variability related to consumption and refill cycles

Storage stability, seasonal variation, and temporal trends

Throughout the study period, Tanks 21/A and 21/B demonstrated excellent operational stability, maintaining nitrogen levels close to maximum capacity. Although greater fluctuations were recorded in Tanks 26/A and 26/B, no prolonged episodes of critically low nitrogen levels were observed. Whenever nitrogen levels dropped below the predefined threshold, refill procedures were promptly performed, ensuring uninterrupted cryogenic preservation. No failures of the cryogenic storage chain and no loss of biological specimens occurred during the study period.

Daily evaporation rates averaged 0.60 units/day for Tank 26/A and 0.68 units/day for Tank 26/B, with no significant overall difference between the two tanks (Mann-Whitney test; p=0.608). Seasonal analysis showed higher evaporation during the warm season than during the cold season. For Tank 26/A, the mean evaporation rate increased from 0.60 units/day during the cold season to 0.79 units/day during the warm season, corresponding to an increase of approximately 32%. For Tank 26/B, evaporation increased from 0.596 to 0.833 units/day, representing an approximately 40% increase that reached statistical significance (Mann-Whitney U=8704.5; p=0.040).

Estimated annual liquid nitrogen consumption reached 219 units for Tank 26/A and 248 units for Tank 26/B, representing an overall annual consumption of approximately 467 units. Linear regression analysis demonstrated a small but statistically significant decline in nitrogen levels over time for Tank 26/A (β=-0.00179 units/day; R²=0.021; p=0.005), whereas no significant temporal trend was observed for Tank 26/B (β=-0.00052 units/day; R²=0.0017; p=0.432). Daily evaporation rates differed significantly across the four years of surveillance for both Tank 26/A (Kruskal-Wallis H=23.87; p<0.001) and Tank 26/B (Kruskal-Wallis H=27.34; p<0.001).

## Discussion

The present study combines a description of institutional sperm cryopreservation activity with a four-year evaluation of routine cryogenic storage quality assurance in a Moroccan university hospital. Oncological indications accounted for most procedures, with testicular tumors and hematological malignancies predominating, while non-oncological indications also contributed substantially. These clinical findings are mainly descriptive and broadly consistent with the existing literature. The more distinctive contribution is the analysis of real-world storage monitoring records, which provides practical data on storage stability, liquid nitrogen evaporation, refill activity, and temporal variation within the unit.

Sperm cryopreservation indications

In our series, oncological diseases represented the primary indication for sperm cryopreservation, with testicular tumors being the most frequent diagnosis, followed by hematological malignancies, particularly leukemia and lymphoma. This distribution is consistent with previous reports identifying testicular cancer and hematological malignancies as the leading indications for fertility preservation in young men because of their high incidence during the reproductive years and the well-established gonadotoxic effects of anticancer treatments [9,10].

Patients with leukemia constitute a particularly vulnerable population regarding future fertility. Previous studies have shown that impaired spermatogenesis may already be present before the initiation of chemotherapy, suggesting that the disease itself may adversely affect testicular function [11,12]. Jayasena et al. further demonstrated that a substantial proportion of newly diagnosed leukemia patients already exhibited severely impaired semen quality at the time of fertility preservation, including a total motile sperm count below one million in a considerable number of patients [13]. Although our study did not compare semen parameters according to cancer type, the presence of several leukemia patients in our cohort reinforces the importance of early referral to fertility preservation services before the initiation of gonadotoxic treatment. In our clinical practice, we also observed that some patients declined sperm cryopreservation, mainly because of treatment urgency or financial constraints. Similar barriers have been reported by Achille et al., who identified inadequate communication between oncologists, patients, and reproductive specialists as one of the major obstacles to fertility preservation [14].

Testicular tumors represented the most frequent indication for sperm cryopreservation in our study. This finding is consistent with previous reports showing that men with testicular germ cell tumors frequently present with impaired sperm concentration and motility even before treatment initiation [15,16]. Shankara-Narayana et al. demonstrated that semen quality was closely associated with total testicular volume and that sperm banking was predominantly performed before gonadotoxic therapy [17]. Other studies have suggested that semen quality may deteriorate after orchiectomy because of reduced functional testicular tissue, oxidative stress, endocrine disturbances, disruption of the blood-testis barrier, and the cumulative effects of cancer therapies [4,18]. Together, these findings support current international recommendations advocating systematic sperm cryopreservation before orchiectomy or any potentially gonadotoxic treatment in patients diagnosed with testicular cancer [4].

Our findings also demonstrate the increasing contribution of non-oncological indications to fertility preservation practice. Patients presenting with severe oligozoospermia, testicular torsion, testicular ectopia, retrograde ejaculation, or an anticipated risk of infertility following surgery underwent preventive sperm cryopreservation. This diversification of indications reflects the progressive evolution of fertility preservation strategies, which are no longer limited to cancer patients but increasingly include men with benign conditions that may compromise their future reproductive potential [19,20].

Several studies have demonstrated that sperm cryopreservation provides men exposed to gonadotoxic treatments with realistic opportunities for future biological fatherhood through assisted reproductive technologies. Reported utilization rates of cryopreserved sperm range from 7% to 30%, with satisfactory reproductive outcomes following in vitro fertilization and intracytoplasmic sperm injection [21]. Our study did not include long-term follow-up of reproductive outcomes or utilization rates of cryopreserved specimens, representing one of its principal limitations. Longitudinal follow-up of our cohort will be essential to evaluate the real impact of fertility preservation on future reproductive outcomes.

The implementation of fertility preservation programs remains highly heterogeneous worldwide. In France, the CECOS network reports that more than 90% of adolescents and young adults diagnosed with germ cell tumors or hematological malignancies are offered sperm cryopreservation before treatment initiation [22]. Similarly, studies from Beijing and several Chinese provinces have documented a progressive increase in referrals for sperm banking over recent years [23,24]. Our experience reflects the ongoing development of fertility preservation services in Morocco, where increasing awareness among oncologists and reproductive specialists appears to be improving access to fertility preservation.

Despite these encouraging developments, several challenges remain. Financial constraints, the urgency of cancer treatment, and the absence of standardized national fertility preservation protocols continue to limit access to sperm cryopreservation in our setting. International guidelines, particularly those issued by the American Society of Clinical Oncology (ASCO), strongly recommend that fertility preservation should be systematically discussed immediately after cancer diagnosis and that eligible patients should be referred promptly to specialized reproductive centers [4]. Strengthening multidisciplinary collaboration among oncologists, hematologists, urologists, and reproductive medicine specialists therefore remains essential to improve access to fertility preservation and optimize the quality of patient care.

Quality assurance of cryogenic storage

To our knowledge, this study is among the first from North Africa to evaluate a quality assurance program for liquid nitrogen storage within a hospital-based sperm cryopreservation unit. Unlike previous experimental studies that investigated induced failures of cryogenic storage tanks, our work was based on routine monitoring data collected under real-life operating conditions, allowing the assessment of storage stability, liquid nitrogen consumption patterns, and the effectiveness of quality assurance procedures over a four-year period.

Our analysis showed that the primary storage tanks remained stable throughout the study period. Liquid nitrogen levels were maintained within the predefined local safety range, and no failure of the cryogenic storage chain or loss of biological specimens was recorded. Within this institutional setting, these observations support the operational effectiveness of regular level monitoring and preventive refilling. They should not, however, be interpreted as proof that the same program will perform identically in other laboratories with different tanks, workloads, environments, or monitoring procedures.

Beyond confirming storage stability, our study provides quantitative evidence on the dynamics of liquid nitrogen consumption under routine laboratory conditions. Daily evaporation rates differed between storage tanks and varied according to operational and environmental conditions. Seasonal analysis demonstrated significantly higher evaporation rates during the warm season for Tank 26/B (p=0.040), whereas no statistically significant seasonal difference was observed for Tank 26/A. Furthermore, the Kruskal-Wallis analysis revealed significant interannual variations in evaporation rates for both tanks (p<0.001), suggesting that liquid nitrogen consumption is influenced not only by ambient conditions but also by operational factors such as storage workload, frequency of tank opening, and the number of cryopreserved specimens.

These observations are consistent with the findings of Pomeroy et al. [25], who demonstrated that the performance of cryogenic storage tanks may progressively change over time and that the liquid nitrogen evaporation rate is one of the most sensitive indicators of tank performance. In their experimental model, deliberate loss of vacuum insulation resulted in a dramatic increase in liquid nitrogen evaporation and a rapid reduction in storage autonomy. Although no comparable structural failure occurred in our laboratory, the variations in evaporation rates observed throughout the monitoring period support the value of longitudinal surveillance as a practical tool for the early detection of abnormal tank behavior before any critical event occurs.

Pomeroy et al. also reported that intrinsic tank characteristics, including tank age, filling level, and internal storage configuration, may substantially influence evaporation performance [25]. Similar mechanisms may explain the differences observed between Tanks 26/A and 26/B in our study despite apparently comparable storage conditions. Variations in storage load, handling frequency, or intrinsic technical characteristics of individual tanks are likely to contribute to the differences in nitrogen consumption observed during routine operation.

Another important observation reported by Pomeroy et al. concerns the limitations of alarm systems based exclusively on temperature monitoring [25]. Their study demonstrated that specimen temperature remains below the glass transition threshold as long as an adequate volume of liquid nitrogen is present within the tank. Once liquid nitrogen is completely depleted, however, temperature rises rapidly, leaving only a limited time for corrective intervention. These findings suggest that direct monitoring of liquid nitrogen levels or evaporation rates may provide earlier detection of storage abnormalities than temperature-based alarm systems alone.

In our laboratory, regular monitoring of liquid nitrogen levels combined with scheduled preventive refilling maintained safe storage conditions throughout the observation period. The evaporation analyses also suggest that longitudinal monitoring of evaporation rates may be a useful additional local quality indicator for identifying unusual increases in nitrogen consumption. Because no tank failure occurred and no external validation was performed, the predictive value and broader applicability of this approach should be evaluated in multicenter and prospective studies.

Study limitations

Nevertheless, our study has several limitations. First, it was conducted at a single center and included only 59 clinical procedures, limiting statistical power and the generalizability of the clinical findings. The clinical analyses were mainly descriptive; the small sample and limited number of events did not support robust multivariable modelling, so potential confounding factors could not be assessed. Second, environmental parameters such as ambient temperature and humidity were not systematically recorded, preventing the direct evaluation of their influence on liquid nitrogen evaporation. In addition, the exact volume of liquid nitrogen added during each refill was unavailable, precluding the accurate estimation of annual consumption in liters. Finally, unlike the experimental study conducted by Pomeroy et al. [25], we did not evaluate alarm system performance or simulate storage tank failures. Consequently, the principal contribution of this study is practical and institution-specific, particularly the longitudinal quality assurance data, rather than definitive evidence of broader clinical effectiveness.

Perspectives

Despite these limitations, our study provides practical data that may contribute to the optimization of cryogenic storage management in assisted reproduction laboratories. Future studies incorporating electronic liquid-level sensors, weight monitoring systems, or real-time remote surveillance technologies may further improve the early detection of abnormal nitrogen consumption, as previously suggested by other authors [26]. Multicenter investigations integrating environmental variables with routine monitoring data would also improve understanding of the factors influencing liquid nitrogen evaporation and facilitate the development of optimized maintenance strategies. Finally, periodic performance evaluation of storage tanks and alarm systems should become an integral component of laboratory quality assurance programs to minimize the risk of specimen loss, in accordance with recommendations issued following major cryogenic storage incidents reported in the literature [27,28].

## Conclusions

This single-center study describes the development of sperm cryopreservation services at Mohammed VI University Hospital. In this cohort, oncological indications, particularly testicular tumors and hematological malignancies, were the predominant reasons for fertility preservation, while non-oncological indications were also represented. These descriptive findings reflect our institutional experience and support timely referral of men at risk of infertility before gonadotoxic treatment or surgery; they should not be generalized beyond comparable settings without further study.

Within the same unit, review of the liquid nitrogen monitoring program showed that safe storage conditions were maintained throughout the study period, with no recorded failure of the cryogenic chain or loss of biological specimens. Seasonal variation in evaporation was identified for one working tank, and interannual fluctuations occurred in both working tanks. These findings support the local use of routine level checks, evaporation rate review, documented refilling, and preventive maintenance as quality assurance indicators, but they do not establish the effectiveness of this program in other institutions.

Taken together, the findings illustrate the operational link between clinical fertility preservation activity and the storage system responsible for safeguarding cryopreserved specimens. Larger multicenter studies with long-term reproductive outcomes, environmental measurements, standardized liquid nitrogen volume data, and automated real-time surveillance are needed to assess generalizability and determine which monitoring indicators best predict storage risk.
